# Effectiveness of the Traditional Japanese Herbal Medicine, Yokuinin (Kampo), in the Treatment of Cervical Precancerous Lesions

**DOI:** 10.7759/cureus.77114

**Published:** 2025-01-07

**Authors:** Chihiro Kiyoshima, Ibuki Kimura, Koko Ishida, Tomoka Hirano, Tomohiro Ishida, Koichiro Shigekawa, Kenichi Yoshikawa, Fusanori Yotsumoto

**Affiliations:** 1 Department of Obstetrics and Gynecology, Faculty of Medicine, Fukuoka University, Fukuoka, JPN

**Keywords:** cervical intraepithelial neoplasia (cin), cervical precancerous lesions, human papillomavirus (hpv), premenopause, yokuinin

## Abstract

Background

Human papillomavirus (HPV) infection precedes cervical dysplasia, culminating in cervical cancer. Yokuinin extract, used for treating verruca vulgaris caused by HPV, exhibits potential antitumor activity against cervical cancer and mild cervical dysplasia. We examined the usefulness of Yokuinin in the treatment of cervical precancerous lesions.

Methods

This retrospective study included 73 female patients with verrucous skin lesions and mildly abnormal cervical cytology diagnosed between April 2019 and August 2021. Of the 73 patients, 34 received Yokuinin treatment whereas 39 did not. The Yokuinin-treated patients received 1.0 g Yokuinin orally divided into three doses daily. Evaluation criteria included retested cervical cytology, time to negative cervical cytology (negative for intraepithelial lesions or malignancies), and side effects. Statistical analyses employed the Mann-Whitney U test and chi-square test, indicating a statistically significant difference (p<0.05).

Results

Yokuinin-treated patients were significantly more likely to achieve normal cytology (NILM) status (p= 0.0441). Median time to NILM was three months for Yokuinin-treated versus five months for non-Yokuinin-treated group, indicating that the Yokuinin-treated group achieved NILM significantly earlier (p= 0.0011). Additionally, high-risk HPV-positive patients were more likely to develop NILM in a short period after Yokuinin administration, and premenopausal patients also developed NILM in a short period. No adverse effects were associated with Yokuinin administration.

Conclusion

Yokuinin administration effectively normalized precancerous cervical lesions, with expedited normalization observed in HPV-positive and premenopausal cases. Yokuinin emerges as a promising treatment for cervical precancerous lesions.

## Introduction

According to GLOBOCAN (Global Cancer Observatory) 2020, cervical cancer affects approximately 604,000 individuals annually worldwide, resulting in 342,000 deaths, with the number of new cases increasing yearly [1]. Recent data indicate a surge in cervical cancer in Japan, particularly among women of reproductive age, especially those in their 20s and 30s [2]. Cervical cancer and its precursor, cervical dysplasia, stem from persistent infection with human papillomavirus (HPV), with primary prevention and early detection strategies revolving around HPV vaccination and cytology-based screening alongside HPV testing [3,4]. Despite recent efforts to promote HPV vaccination and enhance precancerous lesion screening in Japan, uptake rates lag behind those of other countries [5].

Patients diagnosed with cervical low-grade squamous intraepithelial lesions (LSIL), corresponding to mild cervical intraepithelial neoplasia (CIN1), defined as transient HPV infection, are characterized by a high rate of spontaneous CIN 1 regression (approximately 60%). Even if LSIL is detected by screening, there is no effective way to control its progression, as some reports indicate that 10% of cases will progress to HSIL/CIN2-3 [6,7]. Additionally, once the abnormal cytology is detected, even if the progression to HSIL is minimal, the follow-up period can last for years [8], which imposes a small physical and psychological burden on the patient.

A traditional Japanese (Kampo) herbal medicine, Yokuinin, also called adlay (*Coix lachryma-jobi* var. *ma-yuen* Stapf.) seeds have been used to treat viral warts and skin diseases in Japan [9]. Other benefits of adlay include the alleviation of inflammation, endocrine system dysfunction, chapped skin, and neuralgia. Moreover, it is one of the most widely used herbal remedies. Adlay increases natural killer cells and cytotoxic T cells in the peripheral blood and facilitates antiviral activity [10], in addition to its antitumor effects. Consequently, there are increasing reports of its use in clinical practice [11-13]. Many studies have confirmed that Coix seeds and their extracts can reduce the proliferation, invasion, and migration of various cancers, including cervical cancer, and promote apoptosis [14,15]. Although there have been several reports on the efficacy of Coix seed in cervical cancer treatment, no studies have shown its therapeutic effect on cervical dysplasia, a precancerous lesion.

We hypothesized that Yokuinin, which has antiviral and antitumor effects, might inhibit or ameliorate the progression of CIN1 in patients who can wait for regular checkups before CIN1 progresses to cervical cancer. Therefore, this study aimed to clarify the usefulness of Yokuinin extract in patients diagnosed with precancerous lesions of the uterine cervix.

## Materials and methods

This retrospective study was conducted at Fukuoka University, Faculty of Medicine, between April 2019 and August 2021, and included 73 female patients with verrucous skin lesions and mild abnormalities diagnosed by cervical cytology. Patients below the age of 20 were excluded. Clinical and pathological information on the patients was retrospectively obtained from their clinical records. The study protocol received approval from the Institutional Review Board of Fukuoka University Hospital (approval number: U24-06-010). All participants provided verbal informed consent.

Conventional Pap smears were performed on the patients. Cervical smears were classified according to the Bethesda System. Histological diagnoses were made using hematoxylin and eosin-stained sections according to the World Health Organization (WHO) classification. The Pap test was repeated every one to six months. We concluded the follow-up when regression to normal cytology (NILM) was observed on two or more occasions.

Patients treated with Yokuinin were orally administered 1.0 g of Yokuinin extract per day in three divided doses. HPV assays were performed using the Hybrid Capture™ II assay (HC2) (QIAGEN, Inc., Hilden, Germany) or HPV genotyping assay (MEBGEN™ HPV; Luminex Corporation, Austin, Texas, United States) for high-risk HPV (hrHPV). HC2 is a hrHPV test that detects 13 hrHPV types (HPV-16, HPV-18, HPV-31, HPV-33, HPV-35, HPV-39, HPV-45, HPV-51, HPV-52, HPV-56, HPV-58, HPV-59, and HPV-68). MEBGEN™ HPV is an individual-genotyping HPV test that can determine which of the 13 hrHPV types are positive or whether several of them are positive using polymerase chain reaction-reverse sequence-specific oligonucleotide (PCR-rSSO). Patients showing positive results were included in the HPV-positive group, and those with negative results were in the HPV-negative group.

The evaluation of efficacy was based on retest results of cervical cytology and the period from the diagnosis of mild cytological abnormality to regression of NILM. Safety evaluations included assessing the presence or absence of side effects (such as rash, redness, itching, urticaria, stomach discomfort, and diarrhea) caused by Yokuinin administration. Adverse events, including patient-elicited, patient-solicited, and patient-reported symptoms, were graded according to the Common Terminology Criteria for Adverse Events v5.0 (CTCAE v5.0) set by the National Cancer Institute (Bethesda, Maryland, United States).

Statistical methods used included the Mann-Whitney U test and chi-square test, with a significance level set at *p*<0.05 indicating a statistically significant difference. Statistical analyses were performed using GraphPad Prism v9.0 (Insightful Science, LLC, Boston, Massachusetts, United States).

## Results

Patient characteristics

Of the 73 patients, 34 were treated with Yokuinin, and 39 were not. The characteristics of the patients are shown in Table 1. There were no significant differences between the Yokuinin-treated and Yokuinin non-treated groups in terms of age at diagnosis of CIN1 (years), BMI (kg/m2), multigravida, multipara, menopause, and history of allergy and smoking. However, the follow-up period was significantly shorter in the Yokuinin group (p = 0.0007). Upon cytology retest, there was no significant difference between the two groups, but there was a trend toward regression to NILM in the Yokuinin group.

**Table 1:** Characteristics in all groups (n=73) † Data are shown as median (Interquartile range), the Mann–Whitney U test. § Data are shown as n (%), chi-square test. ‡ NILM indicated that no precancerous or cancerous cells were observed in the Pap smear. * indicates a significant difference (*P* < 0.05) “-” indicates data not available. CIN, cervical intraepithelial neoplasia; BMI, body mass index; HPV, human papillomavirus; NILM, negative for intraepithelial lesions or malignancies

-	Yokuinin treated groups (n=34)	Yokuinin non-treated groups (n=39)	Z-value	P-value
Age at diagnosis of CIN1 (years) †	45.0 (36.5-51.25)	38.0 (31-48)	-	0.0548
Follow-up period (months) †	8.5 (4.75-15)	13.0 (10-27)	-	0.0007*
BMI (kg/m2) †	21.55 (19.45-23.9)	20.9 (19.4-22.7)	-	0.2056
Multigravida §	25 (73.5)	25 (64.1)	0.865	0.3871
Multipara §	22 (64.7)	22 (56.4)	0.723	0.4700
Menopause §	11 (32.4)	9 (23.1)	1.711	0.4363
History of allergy §	2 (5.9)	4 (10.3)	0.679	0.4973
Smoking §	1 (2.9)	4 (10.3)	1.234	0.2171
HPV result §	-	-	-	-
HPV-positive (n=32)	17 (50.0)	15 (38.5)	2.520	0.2837
HPV-negative (n=17)	9 (26.5)	8 (20.5)		
no HPV testing (n=24)	8 (23.5)	16 (41.0)		
Cytology results §	-	-	-	-
NILM^ ‡^ (n=47)	26 (76.5)	21 (53.9)	4.879	0.0872
Stable (n=24)	8 (23.5)	16 (41.0)		
Progressive (n=2)	0 (0)	2 (5.1)		

Based on the cervical cytology results, only two cases of progression were observed in the Yokuinin non-treated and HPV-positive groups. Both patients progressed to HSIL and underwent cervical conization, resulting in a final pathology of HSIL/CIN2-3.

The characteristics of HPV types in the HPV-positive group are shown in Table 2. In the Yokuinin-treated group, 14 of 17 cases (82.3%) were hrHPV test-positive, whereas 10 of 15 cases (66.6%) were hrHPV test-positive in the non-treated group. In the individual-genotyping HPV test positive, the Yokuinin-treated group had one case each of HPV-39 (5.9%), HPV-58 (5.9%), and HPV51,52,56,68 (5.9%), whereas the non-treated Yokuinin group had one case of HPV-16 (16.7%), one case of HPV-52 (16.7%), one case of HPV-58 (5.9%), and two cases of HPV-68 (13.3%).

**Table 2:** Characteristics of high risk-HPV type in the HPV-positive groups † High-risk HPV tests targeted the following oncogenic HPV types: HPV-16, HPV-18, HPV-31, HPV-33, HPV-35, HPV-39, HPV-45, HPV-51, HPV-52, HPV-56, HPV-58, HPV-59, and HPV-68. § Individual-genotyping HPV tests can identify one or more of the 13 individual HPV types mentioned. “-” indicates data not available. HPV, human papillomavirus

	Yokuinin treated groups (n=17)	Yokuinin non-treated groups (n=15)
Non-genotyping HPV test^†^ positive (n=24)	14 (82.3%)	10 (66.6%)
Individual-genotyping HPV test^§^ positive (n=8)	-	-
HPV-16	-	1 (6.7%)
HPV-39	1 (5.9%)	-
HPV-52	-	1 (6.7%)
HPV-58	1 (5.9%)	1 (6.7%)
HPV-68	-	2 (13.3%)
HPV-51, 52, 56, 68	1 (5.9%)	-

Efficacy

The cytological retest results are shown in Table 3. Twenty-six out of the 34 patients in the Yokuinin-treated group and 21 out of 39 patients in the non-treated group had NILM, with the two groups being significantly different (p = 0.0441). There was no difference in NILM with Yokuinin administration between menopausal and premenopausal (p = 0.3359 or p = 0.1593, respectively).

**Table 3:** Cytology results for the Yokuinin treated groups and Yokuinin non-treated groups § Data are presented as n (%), chi-square test. ‡ NILM indicated that no precancerous or cancerous cells were observed in the Pap smear. † Non-NILM includes ASC-US (Atypical Squamous Cells of Undetermined Significance) or LSIL (Low-Grade Squamous Intraepithelial Lesion) or HSIL (High-Grade Squamous Intraepithelial Lesion). * indicates a significant difference (*P* < 0.05) “-” indicates data not available. NILM, negative for intraepithelial lesion or malignancy

	Yokuinin treated groups	Yokuinin non-treated groups	Z-value	P-value
All groups (n=73) §	-	-	-	-
NILM ‡ (n=47)	26 (76.47)	21 (53.85)	2.0136	0.0441*
non-NILM † (n=26)	8 (23.53)	18 (46.15)		
Menopause groups (n=20) §	-	-	-	-
NILM (n=14)	9 (81.82)	5 (55.56)	1.2751	0.2023
non-NILM (n=6)	2 (18.18)	4 (44.44)		
Premenopause groups (n=53) §	-	-	-	-
NILM (n=33)	17 (73.91)	16 (53.33)	1.5319	0.1255
non-NILM (n=20)	6 (26.09)	14 (46.67)		

**Figure 1:**
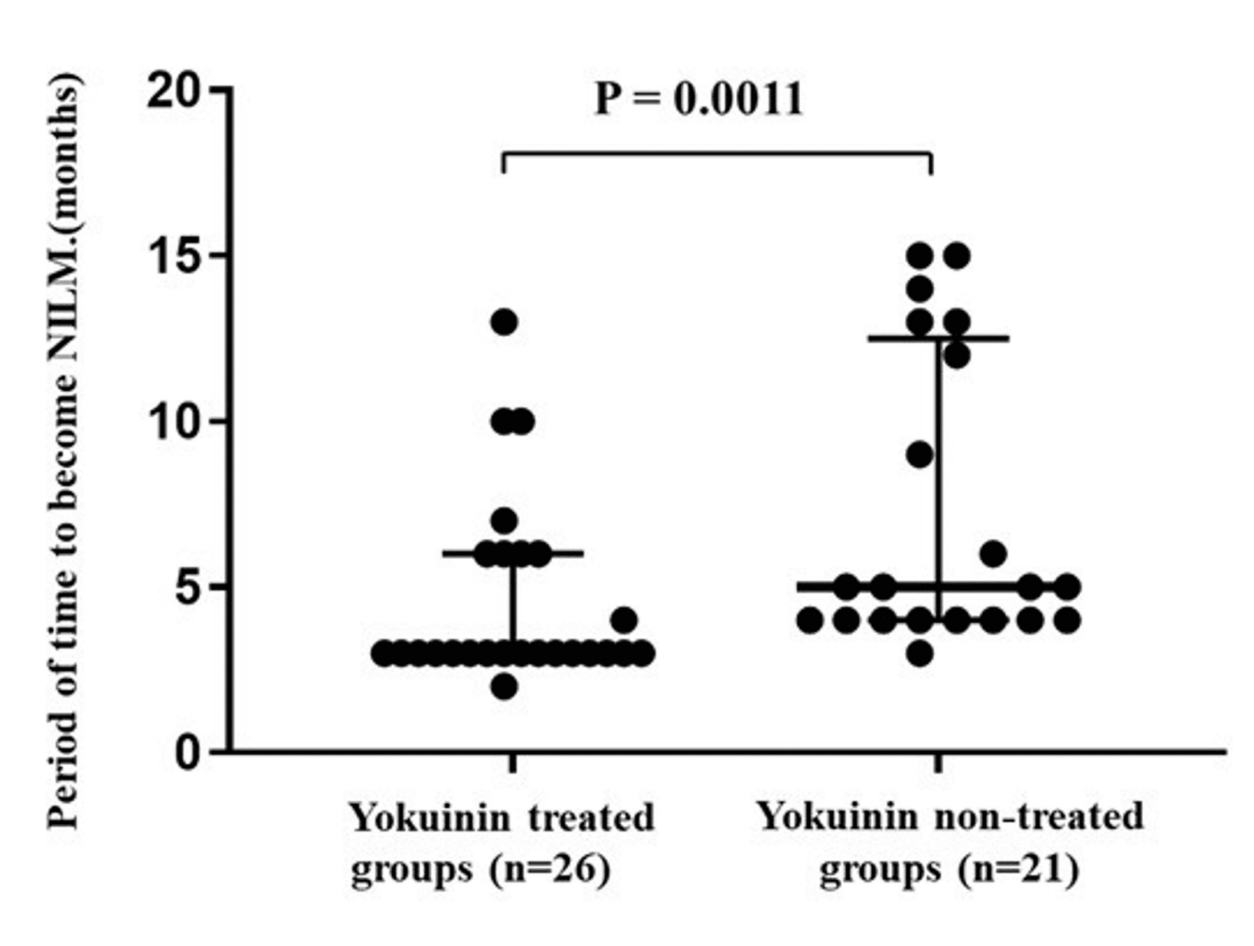
Comparison period of time to become NILM with and without Yokuinin treated in 47 patients with NILM. Black circles indicate the period of time (months) to become NILM in the Yokuinin treated (n=26) and non-treated groups (n=21), respectively. The thick bar indicates the median, and the thin bars indicate the 25th and 75th percentiles, respectively. NILM, negative for intraepithelial lesions or malignancies

Among the 73 patients, 26 in the Yokuinin group and 21 in the non-Yokuinin group developed NILM. The median time to NILM was three months (25th to 75th percentile: 4 to 12.5) in the Yokuinin group and five months (25th to 75th percentile: 3 to 6) in the non-Yokuinin group, which was significantly shorter in the Yokuinin group than in the non-treated group (p = 0.0011) (Figure 1).

**Figure 2:**
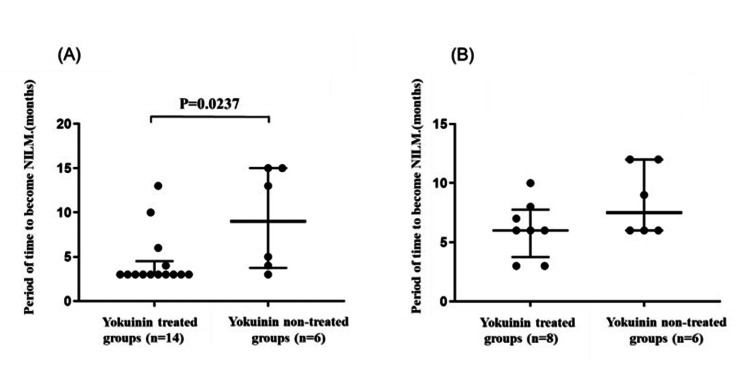
Comparison period of time to become NILM with and without Yokuinin treated in patients with NILM (A) In HPV-positive groups (n=20): Black circles indicate the period of time (months) to become NILM in Yokuinin treated (n=14) and non-treated groups (n=6), respectively. (B) In HPV-negative groups (n=14): Black circles indicate the period of time (months) to become NILM in Yokuinin treated (n=8) and non-treated groups (n=6), respectively. The thick bar indicates the median and the thin bars indicate the 25th and 75th percentiles. NILM, negative for intraepithelial lesions or malignancies

The time to NILM in patients with and without HPV infection was compared between those treated with and without Yokuinin. There was no significant difference in the HPV-negative group (p=0.800). However, NILM occurred significantly more rapidly in the yokuinin group than in the non-treated group in HPV-positive cases (p = 0.0237) (Figure 2).

The median time to NILM in the HPV-positive groups was three months (25th to 75th percentile: 3 to 4.5) in the Yokuinin-treated group and nine months (25th to 75th percentile: 3.75 to 15.0) in the Yokuinin non-treated group (p = 0.0237) (Figure 2A). In contrast, the median time to NILM in the HPV-negative groups was six months (25th to 75th percentile: 3.75 to 7.75) in the Yokuinin-treated group and 7.5 months (25th to 75th percentile: 6.0 to 12.0) in the Yokuinin non-treated group (p = 0.2248) (Figure 2B).

**Figure 3:**
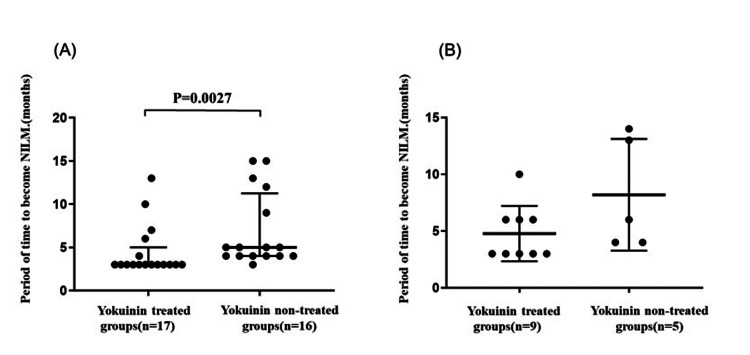
Comparison period of time to become NILM with and without Yokuinin treatment (A) In premenopause groups (n=33): Black circles indicate the period of time (months) to become NILM in yokuinin treated (n=17) and non-treated groups (n=16), respectively. (B) In menopause groups (n=14): Black circles indicate the period of time (months) to become NILM in yokuinin treated (n=9) and non-treated groups (n=5), respectively. The thick bar indicates the median and the thin bars indicate the 25th and 75th percentiles. NILM, negative for intraepithelial lesions or malignancies

Furthermore, the median time to NILM in the premenopausal groups was three months (25th to 75th percentile: 3 to 5) in the Yokuinin-treated group and five months (25th to 75th percentile: 4 to 11.25) in the Yokuinin non-treated group (p = 0.0027) (Figure 3A). Conversely, the median time to NILM in the premenopausal groups was three months (25th to 75th percentile: 3.0 to 6.0) in the Yokuinin-treated group and 6 months (25th to 75th percentile: 4 to 13.5) in the Yokuinin non-treated group (p=0.1104) (Figure 3B).

Safety

No adverse effects were associated with Yokuinin administration. None of the patients reported rash, redness, itching, urticaria, stomach discomfort, or diarrhea at any time during Yokuinin administration. No elicited, solicited, or patient-reported adverse events were observed following Yokuinin administration.

## Discussion

Yokuinin promoted normalization (regressed to NILM) on retest cytology and normalized more quickly than in non-treated cases. Yokuinin was more effective in patients with hrHPV-positive cervical dysplasia, especially among younger, premenopausal patients. Yokuinin demonstrated no side effects and showed good adherence due to its lack of subjective symptoms and slightly sweet taste, making it easy for patients to consume regularly.

This study supports the notion that Coix seed (Yokuinin) has an antitumor effect not only on cervical cancer but also on its precancerous lesions. Although there are no reports on the effects of Coix seeds on precancerous lesions, there are several reports on their antitumor effects in cervical cancer. One study reported that dissolving paclitaxel in Coix seed oil synergistically helped fight cancer, exerted stronger in vitro cytotoxicity, and induced cell apoptosis, which had a stronger therapeutic effect on cervical cancer [16]. Additionally, one report stated that the joint application of Coix seed oil and tripterin can work synergistically on the proliferation of cervical cancer, anti-angiogenesis, and induction of cell apoptosis [17]. Coix seed is an important food and component of traditional Chinese medicine in China, Japan, and other Asian countries, and it is a commonly used clinical drug with antitumor, immune regulation, hypoglycemic, anti-inflammatory, intestinal microbiota improvement, serum lipid-lowering, and angiogenesis promoting effects [15] Therefore, such synergistic effects may have resulted in faster normalization of cervical precancerous lesions.

The mechanism by which HPV-positive patients normalize more quickly after Yokuinin administration may be due to its antiviral and antitumor effects. One report found that the ratio of CD16+CD57- cells, which have the strongest NK activity, to the unique subset of CD3+CD56- cells, which are T cells that can cause cytotoxicity in an MHC-unrestricted manner, increased with Yokuinin treatment [10]. Alternately, Yokuinin increases the number of NK cells and cytotoxic T cells in peripheral blood, resulting in antiviral effects. There have been no direct reports on the suppression of HPV infection by Coix seeds. Notably, Coix seeds are currently used as a traditional medicine for COVID-19 treatment in China [18]. In addition, adlay tea (Coix seed tea) at a concentration suitable for drinking inhibited the multiplication of influenza viruses. Individual components of the tea had antiviral activities against the influenza A/PR/8/34 virus, and adlay tea inhibited influenza infection during periods of virus adsorption to the cell and virus replication [19]. These reports indicate that it is highly likely that Coix seeds can suppress HPV.

Yokuinin also normalized in a short period in younger women. Coix seed has been reported to exhibit higher antioxidant capacity than vitamins C and E, and has a linear relationship with antimelanin activity [20]. Some reports suggest that astragalus has a protective effect on the skin after radiation therapy and that this protective effect is due to its free radical scavenging, antioxidant, anti-inflammatory, wound healing, and skin protective effects [21]. These reports indicate that, from a Chinese medicine perspective, Coix seeds can promote skin turnover and so-called healthy skin. We hypothesized that these mechanisms may result in faster normalization in young women with a faster turnover.

The present study demonstrates that Yokuinin administration is beneficial for treating cervical precancerous lesions by inhibiting their progression and improving them synergistically, especially in HPV-positive and young women. The significance of this study is that it demonstrated the possibility of a noninvasive therapeutic approach for managing precancerous lesions of the cervix in women of reproductive age, providing an alternative to monitoring until progression. However, owing to the retrospective nature and small sample size of this study, future research with a larger sample size, a prospective design, and ideally a randomized controlled trial is needed to provide more robust and accurate data. Additionally, efforts should focus on developing improved management strategies, including the use of Yokuinin alongside HPV vaccination recommendations and widespread cervical cytology screening, to help eradicate cervical cancer.

## Conclusions

Normalization of cervical precancerous lesions was achieved earlier in patients treated with Yokuinin than without Yokuinin, and even earlier in HPV-positive or premenopausal patients. Yokuinin administration appears to be beneficial for the treatment of precancerous lesions of the cervix.
